# Blood plasma metagenomic next-generation sequencing for identifying pathogens of febrile neutropenia in acute leukemia patients

**DOI:** 10.1038/s41598-023-47685-6

**Published:** 2023-11-20

**Authors:** Yan Qi, Wu-Qiang Lin, Bin Liao, Jia-Wei Chen, Ze-Song Chen

**Affiliations:** 1 Department of Hematology, The Affiliated People’s Hospital of Fujian University of Traditional Chinese Medicine, Fuzhou, Fujian China; 2 Department of Hematology, The First Hospital of Putian City, Putian, Fujian China

**Keywords:** Diseases, Medical research

## Abstract

To investigate the value of metagenomic next-generation sequencing (mNGS) in acute leukemia (AL) patients with febrile neutropenia (FN). We retrospectively reviewed 37 AL patients with FN and compared the results of mNGS with blood culture (BC) and the clinical features of the mNGS-positive group and the mNGS-negative group. A total of 14 detected pathogens were the final clinical diagnosis, of which 9 strains were detected only by mNGS and 5 strains were detected by both mNGS and BC. The top pathogens were *Klebsiella pneumoniae*, *Pseudomonas aeruginosa* and *Stenotrophomonas maltophilia*. A total of 67.57% (25/37) were bacterial infections, and 2.7% (1/37) were fungal or viral infections. The diagnostic positivity rate of mNGS (25/37, 67.6%) was significantly higher than that of BC (7/37, 18.9%), and the difference was statistically significant (p < 0.05). Then, we explored the clinical distinction between the mNGS-positive group and the mNGS-negative group, and 3 features were filtered, including lymphocyte count (LY), creatinine levels (Cr), and white blood cell count (WBC). Our study demonstrated that early implementation of mNGS can effectively improve the efficacy of pathogen detection in AL patients with FN. The higher diagnostic positivity rate and the ability to detect additional pathogens compared to BC made mNGS a valuable tool in the management of infectious complications in this patient population. Furthermore, the identified clinical features associated with mNGS results provided additional insights for the clinical indication of infection in AL patients with FN.

## Introduction

Febrile neutropenia (FN) is a common complication in patients with acute leukemia (AL) and poses significant challenges in terms of prompt and accurate diagnosis of the causative pathogens^1^. More than 80% of patients with hematopoietic malignancies will develop neutropenia-related fever after ≥ 1 course of chemotherapy^2^. Due to the low immune function of patients with FN, the symptoms and signs of inflammation are often not obvious, fever may be the only sign of infection, and the pathogen and infection foci are also unclear^3^. Among those patients with FN who received antibiotic treatment at the time of sampling, less than 15% were able to identify infectious microorganisms by blood culture. The failure to identify pathogens may contribute to the overuse or misuse of antimicrobial agents and poor patient outcomes. Additional tools are thus needed for the diagnosis of infection.

Metagenomic next-generation sequencing (mNGS) is a promising approach that enables the detection of nearly all known pathogens directly from clinical samples. However, published reports describing the usefulness of mNGS in AL patients with FN are limited^4,5^. The question remains whether the diagnostic performance and yield of mNGS of plasma microbial cell-free DNA (mcfDNA) justifies its wider adoption for AL patients with FN by the medical community. Therefore, we conducted a retrospective study to assess the value of mNGS in AL patients with FN. We aimed to investigate the value of mNGS in AL patients with FN by comparing its results with those of blood culture (BC) and evaluating the clinical features of mNGS-positive and mNGS-negative groups.

## Results

### Patient characteristics

Between February 2020 and February 2023, a total of 48 patients were screened and referred for review and enrollment in this study (Table 1). We excluded 7 patients who disagreed to undergo mNGS testing, 3 patient whose clinical information was incomplete and 1 patient co-infected with HIV. A total of 37 AL patients with FN were enrolled, including 18 males (48.6%) and 19 females (51.4%). The mean age was 50.3 ± 14.6 years old, and 62.2% of patients were older than 46 years old (46–75 years old, 23/37). The most common leukemia type was acute myeloid leukemia (AML, 26/37, 70.3%), followed by acute lymphoblastic leukemia (ALL, 5/37, 13.5%), acute monoblastic leukemia (AMOL, 3/37, 8.1%), acute hyperleukocytic leukemia (AHL, 2/37, 5.4%) and acute leukemia (AL, 1/37, 2.7%). Among them, 22 patients (59.5%) had focal infections, 13 patients (35.1%) had respiratory infections, 5.4% of patients had urinary tract infections (2/37) and skin infections (2/37), and 2.7% of patients had digestive tract infections (1/37) and perianal infections (1/37). The mean length of stay in the hospital was 42.7 ± 21.4 days, and the median length of stay was 38(18, 125) days.

**Table 1:** Patient characteristics.

Characteristic	No. (%)
Total participants	37 (100%)
Age	
Mean	50.3 ± 14.6
Median (min, max)	49 (20, 69)
Distribution—no. (%)	
15–45	14 (37.8%)
46–75	23 (62.2%)
Male sex—no. (%)	18 (48.6%)
Leukemia type—no. (%)	
AML	26 (70.3%)
ALL	5 (13.5%)
AMOL	3 (8.1%)
AHL	2 (5.4%)
AL	1 (2.7%)
Focal infection—no. (%)	
No	15 (40.5%)
Yes	22 (59.5%)
Respiratory infection	13 (35.1%)
Urinary tract infection	2 (5.4%)
Skin infection	2 (5.4%)
Digestive tract infection	1 (2.7%)
Perianal infection	1 (2.7%)
Others infection	3 (8.1%)
Length of stay (range)-days	
Mean in hospital	42.7 ± 21.4
Median in hospital (min, max)	38 (18, 125)

### Performance of blood mNGS relative to BC

**Figure 1:**
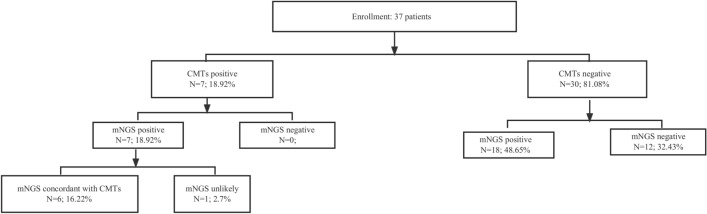
Overall results for BC and mNGS in 37 AL patients with FN.

**Figure 2:**
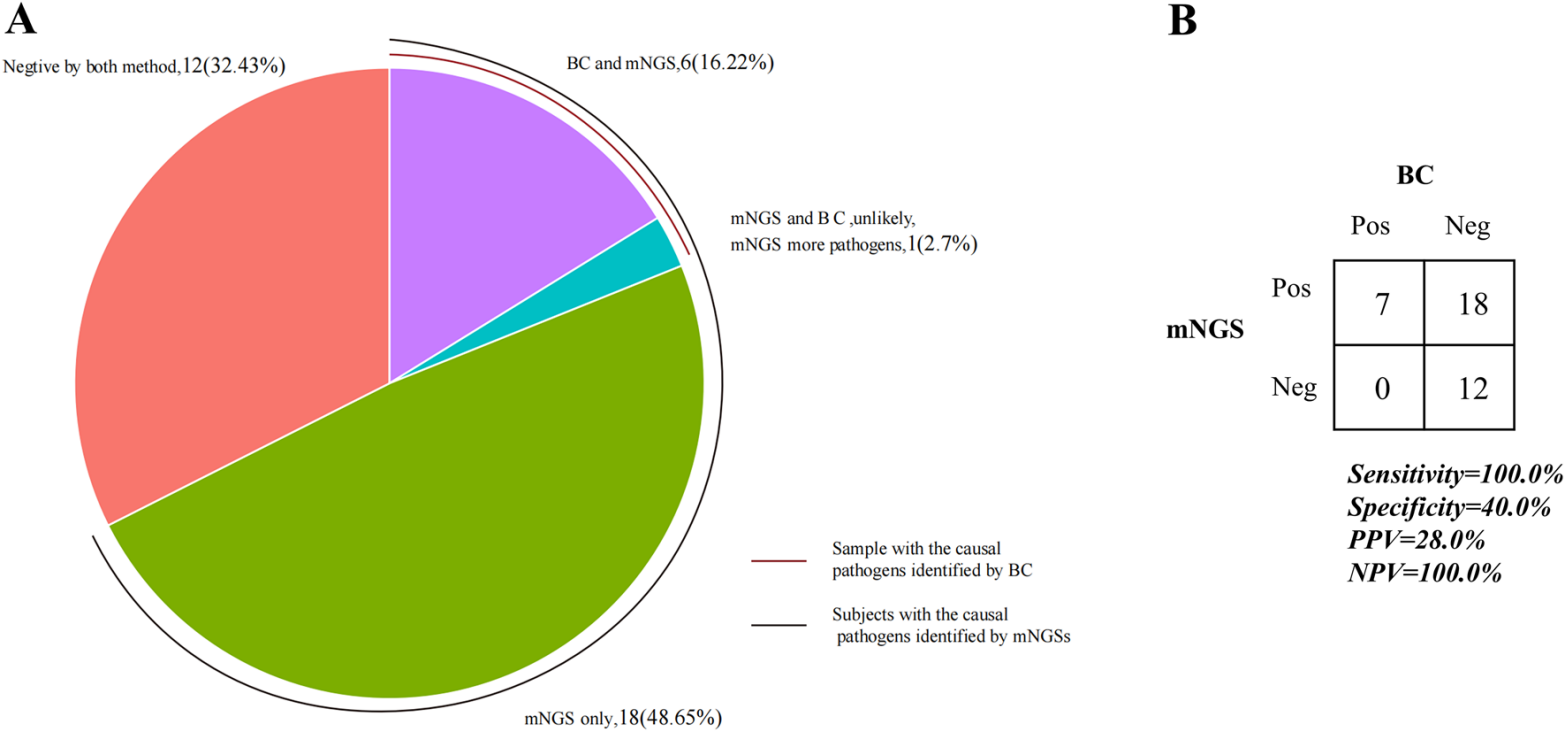
Assay performance of mNGS. (**A**) The panel shows the proportion of samples with pathogens identified by different methods. (**B**) The panel is the 2 × 2 contingency tables comparing the performance of mNGS relative to BC.

**Figure 3:**
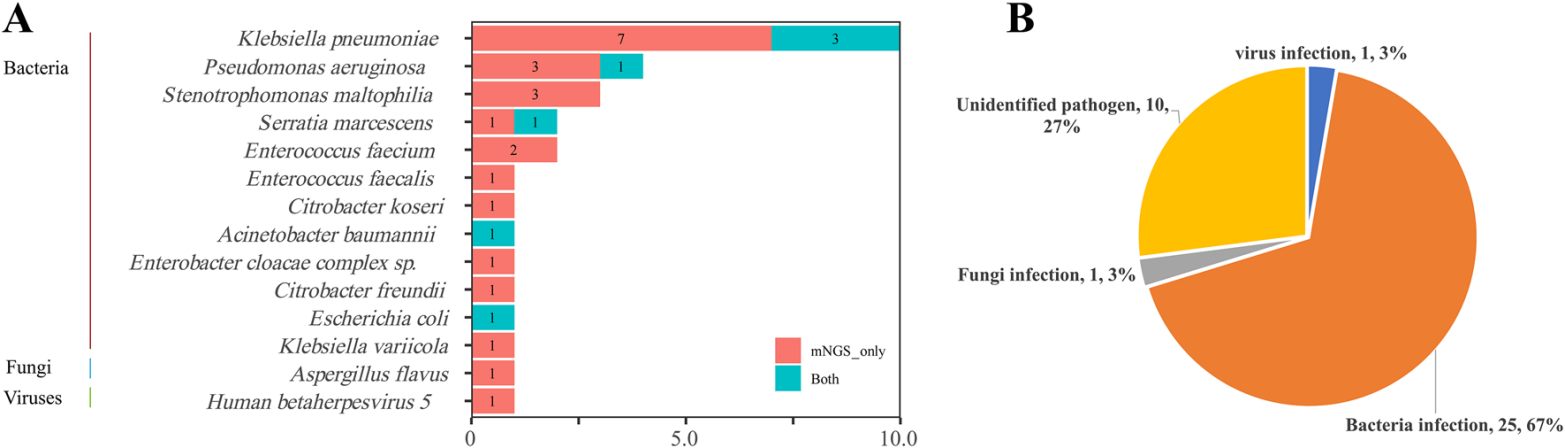
Infection profiles of AL patients with FN. (**A**) Distribution of major pathogens identified in patients using BC and mNGS. Bacteria, fungi and viruses with higher frequencies were selected separately and are shown in the bar chart. mNGS_only, only mNGS one method. Both, BC and mNGS methods. (**B**) Distribution of infection types.

Among 37 AL patients with FN, the diagnostic positivity rate of mNGS (67.6%, 25/37) was significantly higher than that of BC (18.9%, 7/37), highlighting the superior sensitivity of mNGS in pathogen detection (Fig. 1). Of 37 infections, 6 (16.22%) were diagnosed by both BC and mNGS, 18 (48.65%) by mNGS only, and 12 (32.43%) were negative by both BC and mNGS (Fig. 2A). Then, we assessed the clinical performance of mNGS and BC according to the final clinical diagnosis in blood samples. As shown in Fig. 2B, the agreement value of mNGS and BC was 51.35% (19/37). The sensitivity and specificity were 100.00% and 40.00%, respectively. The positive prediction value (PPV) and negative prediction value (NPV) were 28.00% and 100.00%, respectively. Furthermore, we compared the detected and clinically approved pathogens by BC and mNGS (Fig. 3A), including 12 bacteria, 1 fungus and 1 virus. The top 5 detection pathogens were *Klebsiella pneumoniae* (n = 10), *Pseudomonas aeruginosa* (n = 4), *Stenotrophomonas maltophilia* (n = 3), *Serratia marcescens* (n = 2) and *Enterococcus faecium* (n = 2). The pathogens consistently detected by both BC and mNGS were *Klebsiella pneumoniae*, *Pseudomonas aeruginosa*, *Serratia marcescens*, *Acinetobacter baumannii* and *Escherichia coli*. The detailed detected pathogens are shown in Table S1. Most AL patients with FN had bacterial infections (25, 67.57%), 1 was infected with fungi (2.70%), 1 was infected with a virus (2.70%) and 10 had no infections (27.30%, Fig. 3B).

### Clinical features of infection in AL patients with FN with positive mNGS results

**Figure 4:**
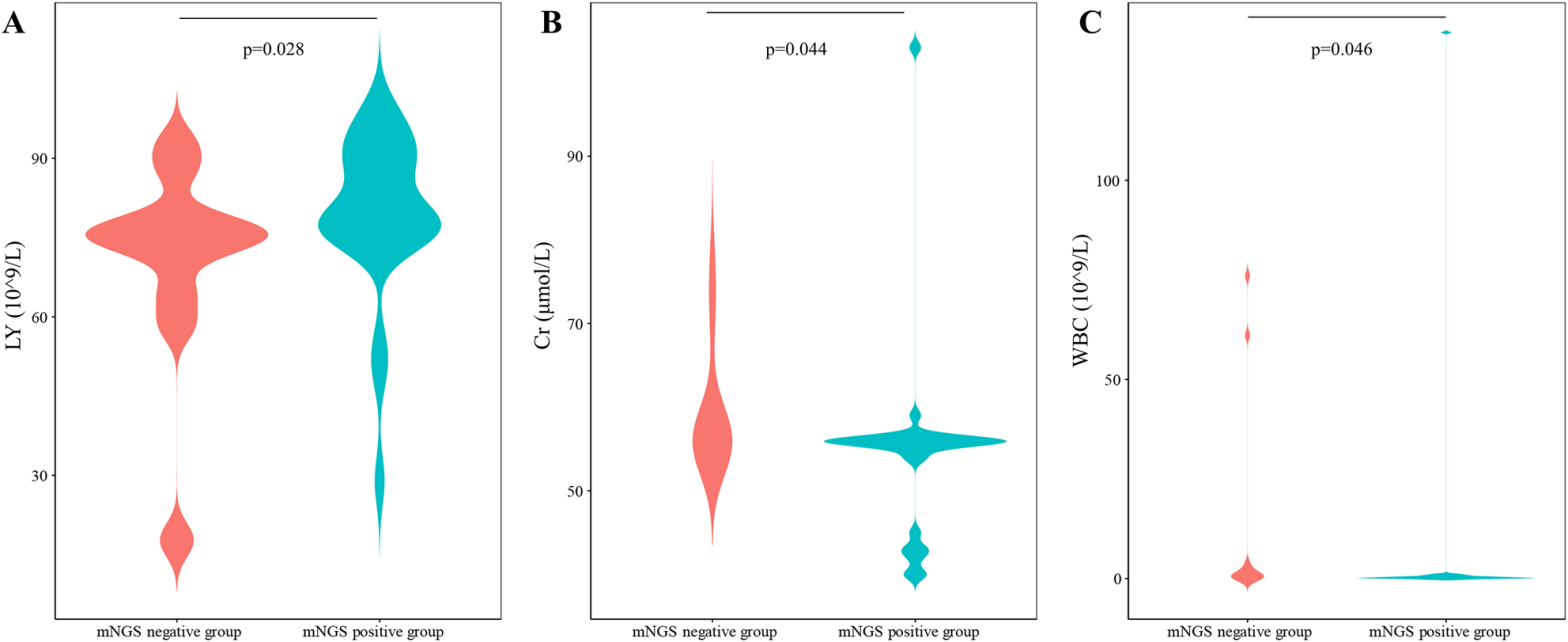
Clinical indication for mNGS application. Comparison of three clinical indicators, LY (**A**), Cr (**B**) and WBC (**C**), between the mNGS-positive group and the mNGS-negative group using the Wilcoxon rank sum test. LY, lymphocyte count. Cr, creatinine levels. WBC, blood white blood cell count. p value < 0.05 indicated a statistically significant difference.

To provide additional insights for the clinical indication of infection in AL patients with FN, we investigated the clinical features between the mNGS-positive groups and mNGS-negative groups. Three features, namely, lymphocyte count (LY), creatinine levels (Cr), and white blood cell count (WBC), were filtered and were significantly different between the two groups (Fig. 4). Compared with the mNGS-negative group, LY increased significantly in the mNGS-positive group (70.39 ± 19.82 vs. 79.42 ± 15.59 10^9^/L, p = 0.028), Cr decreased significantly in the mNGS-positive group (59.20 ± 7.44 vs. 54.53 ± 11.55 μmol/L, p = 0.044), and WBC was reduced significantly in the mNGS-positive group (13.27 ± 27.57 vs. 5.58 ± 26.84 10^9^/L, p = 0.046). The details of the comparison of clinical features are shown in Table 2.

**Table 2:** Clinical features of infection in AL patients with FN with positive mNGS results.

Items	Mean (P)	SD (P)	Mean (N)	SD (N)	p value
LY (10^9^/L)	79.42	15.59	70.39	19.82	0.028
Cr (μmol/L)	54.53	11.55	59.20	7.44	0.044
WBC (10^9^/L)	5.58	26.84	13.27	27.57	0.046
PCT (ng/mL)	0.52	0.78	0.17	0.25	0.104
NEUT (10^9^/L)	14.34	9.15	21.65	20.40	0.150
CRP (mg/L)	29.15	14.48	23.19	9.32	0.217
LDH (U/L)	147.82	78.34	137.43	16.60	0.750
ALB (g/L)	35.04	1.89	34.91	1.58	1.000

LY, lymphocyte count. Cr, creatinine levels. WBC, blood white blood cell count. PCT, procalcitonin. NEUT, neutrophil count. CRP, C-reactive protein. LDH, lactate dehydrogenase. ALB, albumin. avg, average. sd, standard deviation. P, mNGS-positive group. N, mNGS-negative group.

## Discussion

Febrile neutropenia is a serious condition that often affects patients with acute leukemia, compromising their immune system and leaving them highly susceptible to infections. Identifying the causative pathogens responsible for febrile neutropenia is crucial for appropriate and timely treatment selection^6,7^. The precise diagnosis of microbial infections and timely antibiotic therapy are critical to the clinical care of immunocompromised patients. However, conventional microbiologic assays with limited broad pathogen coverage exhibit a low positive rate and are time-consuming^8^. In this study, we conducted a retrospective cohort of 37 AL patients with FN to assess the value of mNGS in this disease. The mean age was 50.3 years old, and 62.2% of patients were older than 46 years old (46–75 years old, 23/37). A total of 59.5% of patients had focal infections, and 35.1% of patients had respiratory infections. The mean length of stay in the hospital and median length of stay were 42.7 days and 38 days, respectively. The rapid and accurate diagnosis of pathogens still presents challenges.

In recent years, metagenomic next-generation sequencing (mNGS) of blood plasma has emerged as a promising tool for unbiased pathogen identification in various infectious diseases^9,10^. mNGS offers several advantages that conventional detection cannot provide. mNGS can identify and detect an infinite range of organisms in a single test, unaffected by genomic diversity and mutations, without preselection of pathogens of interest, and independent of organism culture^11–13^. The positive detection rate of the mNGS test was significantly higher than that of blood culture. For pathogen testing, mNGS is more suitable for bacterial testing, especially for gram-negative bacteria such as *Escherichia coli*, *Klebsiella pneumoniae*, *Pseudomonas aeruginosa*, and *Enterobacter cloacae complex *sp. These bacteria could cause many diseases. Yu et al.^14^ illustrated that the main pathogenic bacteria were *Escherichia coli* and *Klebsiella pneumoniae*, which cause community-acquired infections and nosocomial infections, respectively. Another article proved that the incidence of nosocomial bloodstream infection caused by *Escherichia coli* and *Klebsiella pneumoniae* increased in a tertiary referral center in Taiwan^15^. Moreover, several studies using cfDNA analysis of pathogens in blood to diagnose deep-seated infections have been reported recently^8,16–18^. Although the detection of microbial cfDNA in serum specimens may be indicative of bloodstream infection with worse clinical outcomes in patients, short fragments of DNA do not necessarily imply the presence of live pathogens^19^. Clinicians need to combine the patient’s clinical symptoms to determine the occurrence of bloodstream infection. The pathogens reported in this paper were all detected and clinically approved based on the comprehensive judgment of three clinicians. In this study, the most commonly detected pathogens were *Klebsiella pneumoniae*, *Pseudomonas aeruginosa*, *Stenotrophomonas maltophilia*, *Serratia marcescens* and Enterococcus faecium. The diagnostic positivity rate of mNGS (25/37, 67.6%) was significantly higher than that of BC (7/37, 18.9%). Furthermore, the sensitivity and specificity of mNGS were 100.00% and 40.00%, respectively. The validity of mNGS is given according to BC. In this case, sensitivity was reported to be higher and specificity lower than in reality. It should be emphasized that the 40% specificity of mNGS may be due to the fact that some infections are missed with BC rather than the high number of false positives. The PPV and NPV were 28.00% and 100.00%, respectively. Thus, mNGS testing enables faster and more accurate detection of these pathogens and demonstrates good clinical performance. Therefore, it is certainly a good diagnostic candidate for severely ill patients.

In this study, the use of mNGS demonstrated superior performance compared to BC in detecting pathogens associated with febrile neutropenia. The diagnostic positivity rate of mNGS was significantly higher, indicating its enhanced sensitivity and ability to identify a broader spectrum of pathogens. Importantly, mNGS detected a substantial number of infections that would have been missed by BC alone. This highlights the potential of mNGS to provide a more comprehensive and unbiased assessment of the pathogen landscape in febrile neutropenia patients, enabling improved diagnostic accuracy and more targeted treatment.

The advantages of mNGS are twofold. First, mNGS allows for the simultaneous sequencing of nucleic acids from all organisms present in a sample, offering a comprehensive view of the microbial community. This unbiased approach avoids the need for knowledge of specific pathogens a priori and enables the identification of both common and rare pathogens, including bacteria, fungi, viruses, and even parasites. Thus, mNGS has the capability to detect pathogens that may be challenging to identify using traditional culture-based methods.

Second, mNGS exhibits superior sensitivity compared to BC, as demonstrated by the higher diagnostic positivity rate. Its ability to detect a higher number of infections, particularly those missed by BC, provides a valuable diagnostic advantage. This is especially important in the context of febrile neutropenia, where timely and accurate identification of pathogens is crucial for guiding appropriate treatment decisions. The comprehensive pathogen detection offered by mNGS can inform clinicians about the specific pathogen causing the infection, allowing for targeted antimicrobial therapy and reducing the reliance on empirical broad-spectrum antibiotics.

Moreover, the analysis of clinical features associated with positive mNGS results further supports the clinical feasibility and utility of this technique. The observed differences in LY, Cr, and WBC highlight the potential of these parameters as clinical markers for infection in febrile neutropenia patients. Monitoring these features alongside mNGS results could aid in the early identification of infections and guide treatment decisions.

Nevertheless, there were some deficiencies in our study. Firstly, the limited sample size may affect the accuracy of the experimental study. Secondly, both BC and mNGS lack unified standards to identify whether detected pathogenic microorganisms are derived from infection, colonization, or contamination. For practical applications of this technique, the subjective judgment of clinicians is still needed, which is highly dependent on clinical experience. Although consensus was reached in this study by three experienced senior physicians and was based on the clinical manifestations of patients combined with other laboratory results, subjective bias is still possible.

## Materials and methods

### Subjects and study design

From February 2020 to February 2023, a total of 37 AL patients with FN were enrolled in the Affiliated People’s Hospital of Fujian University of Traditional Chinese Medicine. The inclusion criteria were as follows: (1) age > 18 years; (2) presence of hematological diseases confirmed by bone marrow aspiration, having received pharmacological chemotherapy, immunosuppressive agents, or glucocorticoids; and (3) meeting the criteria for FN: oral temperature > 38.3 °C or twice consecutive > 38.0 °C for more than 1 h and neutrophil count < 500/mm^3^^20^; (4) Blood samples were collected and sent for both BC and mNGS either simultaneously or with an interval of less than 24 h. The exclusion criteria were as follows: (1) those with combined primary immunodeficiency or human immunodeficiency virus (HIV), (2) those with drug-induced fever, (3) those with incomplete medical records, and (4) those who did not undergo mNGS. Patient information was extracted from their medical records, including age, sex, underlying diseases, etc. Clinical indicators tested during the first 24 h after admission were recorded. Treatment strategies and related changes during follow-up were recorded. All patients signed informed consent forms, and this study was approved by the ethics committee of the hospital. I confirm that all the methods were conducted following the relevant guidelines and regulations.

### Data collection

The patient's electronic medical record provided the baseline information that was gathered. demographic traits, such as sex, age, and underlying illnesses. In addition to symptoms and signs, leukemia type, infection location, and length of stay were also part of the medical history. BC was performed in the microbiological laboratory. Other tests included imaging examination of the infection site and infection-relevant indicators such as LY, Cr, WBC, PCT, NEUT, CRP, LDH and ALB. Three experienced hematology doctors examined these data.

### Specimen collection

Blood was collected from all patients through blood draw. Samples were sent to Genskey Co., Ltd. (Beijing, China) for sequencing as described in detail below. The remaining specimens were sent to our microbiological laboratory for BC.

### Metagenomic next-generation sequencing

Blood samples (5 mL) were collected using K2-EDTA tubes (Becton Dickinson, Franklin Lakes, NJ) and centrifuged at 1600 × g for 10 min. The plasma supernatant was used for cfDNA extraction. After centrifugation of blood, 600 μL supernatant was taken, and DNA was extracted using a microsample genomic DNA extraction kit (1901, Genskey, Tianjin). The DNA libraries were constructed by DNA enzyme digestion (200–300 bp), end repair, a-tailing, adapter ligation, and PCR amplification using an NGS library construction kit (2012B, Genskey, Tianjin). The quality of the DNA libraries was assessed using an Agilent 2100 Bioanalyzer (Agilent Technologies, Santa Clara, USA) combined with qPCR to measure the adapters before sequencing. The single-stranded circular DNA was added by 2–3 quantitative sets to obtain DNA nanospheres. The DNA nanospheres were loaded on the sequencing chip and sequenced using the MGISEQ-2000 sequencing platform (MGI, Shenzhen, China). For each run, we used an environmental control sample (human nucleic acid) to monitor microbial DNA signals arising from the background at the time of batch processing and used different ID spike variants (Arabidopsis-specific fragments) to monitor sample-to-sample contamination.

### Bioinformatic analysis of mNGS

For quality control adapter contamination and low-quality and low-complexity reads, raw reads were filtered by fastp (v0.19.5)^21^ and Komplexity v0.3.6^22^. Reads that were mapped to the human reference assembly GRCh38 were removed with Bowtie2 v2.3.4.3^23^. Then, reads were aligned to the microorganism database consisting of approximately 12,000 genomes with SNAP v1.0 beta.18^24^ as previously described^25^. The mapped reads were classified based on the NCBI RefSeq genome database, or the NCBI GenBank genome database was selected for each species. After filtering false positive organisms^26^, we counted the species or genus abundance with Perl scripts.

### Criteria for the mNGS results, discharge diagnosis and pathogen consistency

For all pathogens originally detected, the obvious sequence alignment abnormalities (for the detected species, genome coverage < 1% and depth < 2) and background polluted bacteria (considered to be the exact background bacteria when the taxon-specific read number falls within the normal fluctuation range of historical statistical data compared with the negative controls) were first filtered out^27^. Pathogen data interpretation and pathogen positive determination were then carried out. The details of diagnostic performance of mNGS pathogen determination was shown in Supplementary Materials.

### mNGS clinical performance

The definition and calculation rules of mNGS clinical performance, including sensitivity, specificity, positive predictive value (PPV), negative predictive value (NPV) and diagnostic agreement rate, are shown in Table S1. The final diagnosis of the infectious pathogen at the time of discharge was the gold standard.

### Data available

Sequencing data (with human reads removed) are available from PRJNA949987 in the NCBI Sequence Read Archive (SRA).

### Statistical analysis

The patient characteristics data were normally distributed and expressed as the mean standard deviation (SD). Nonnormally distributed data are described by the median and interquartile range (IQR). For patients with clinical indicator data labeled as no detected or missing, the missing values were imputed by assigning the average value of that indicator from the available data. Comparative analysis was conducted by Wilcoxon rank-sum test in R (version 3.6.3). p values of < 0.05 were considered significant. All statistical analyses and drawings were performed using ggpubr or ggplot2 package in R language (version 3.6.3).

### Ethical approval

This study was approved by the Medical Ethics Committee of the Affiliated People’s Hospital of Fujian University of Chinese Medicine.

## Conclusion

In conclusion, blood plasma mNGS holds significant promise in the management of febrile neutropenia in acute leukemia patients. Its unbiased and comprehensive approach to pathogen detection, coupled with its superior sensitivity, makes it a valuable tool for accurate and timely identification of causative pathogens. The integration of mNGS into clinical practice has the potential to enhance diagnostic accuracy, optimize treatment strategies, and contribute to improved patient outcomes in febrile neutropenia. Further research and validation are needed to establish standardized protocols and guidelines for the routine use of mNGS in this clinical setting.

### Supplementary Information

Supplementary Information.

## Data Availability

The data that support the findings of this study are available from the corresponding author upon reasonable request.
