# Supplementary material for: Blood plasma metagenomic next-generation sequencing for identifying pathogens of febrile neutropenia in acute leukemia patients
Source: Sci Rep. 2023 Nov 20;13:20297. doi: 10.1038/s41598-023-47685-6 (PMC10662164; doi:10.1038/s41598-023-47685-6)
Supplement: Supplementary file 1 — Supplementary Information. [file 41598_2023_47685_MOESM1_ESM.doc]

**Supplementary Appendix**

This appendix has been provided by the authors to give readers additional information about their work.

Blood Plasma Metagenomic Next-generation Sequencing for Identifying Pathogens of Febrile Neutropenia in Acute Leukemia Patients

Yan Qi ^1¶^*, Wu-Qiang Lin ^2¶^, Bin Liao ^1^, Jia-Wei Chen ^1^, Ze-Song Chen ^1^

^1^ Department of Hematology, The Affiliated People’s Hospital of Fujian University of Traditional Chinese Medicine, Fuzhou, Fujian, China

^2^ Department of Hematology, The First Hospital of Putian City, Putian, Fujian, China

***Correspondence:**

Corresponding Author: Yan Qi

E-mail: [**qiydoctor@163.com**](mailto:qiydoctor@163.com)

**TABLE OF CONTENTS**

[**Supplementary Methods 4**](#__RefHeading___Toc143607268)

[Diagnostic performance of mNGS pathogen determination 4](#__RefHeading___Toc143607269)

[Table S1. The definition and calculation rules of mNGS clinical performance. 5](#__RefHeading___Toc143607270)

[Table S2. Clinical features of infection in AL patients with FN with positive mNGS results. 6](#__RefHeading___Toc143607271)

# Supplementary Methods

## Diagnostic performance of mNGS pathogen determination

For clinical adjudication, each of 3 clinical specialists independently classified pathogens identified by NGS according to the likelihood that a pathogen caused the FN episode[^1^](#_ENREF_1). Adjudication cases were classified as “definite”, “probable”, “possible”, “unlikely”, “false negative”, “true negative”, and “indeterminate”. “Definite” is defined as that mNGS pathogen result is concordant with at least one pathogen identified on CMT performed within 7 days of mNGS sample collection and is a likely cause of FN. “Probable” is defined as the results of mNGS and CMT are discordant, mNGS-test pathogen result is a likely cause of FN based on clinical, radiologic, or laboratory findings. “Possible” means that mNGS and CMT results are discordant, mNGS result is consistent with an infection but not a common cause, based on adjudicators’ clinical experience, and available literature, on FN. Diagnosis must be made using history, examination, and non-microbiologic testing and no other focal infection has been reported. “Unlikely” means that mNGS is positive and discordant with microbiological tests and/or not a plausible cause of infection, or there is a more likely explanation for the febrile neutropenic event not meeting “possible” or “probable” classification criteria. When the mNGS is negative while microbiologic tests are positive and adjudicated as the cause of infection or results from other microbiologic tests are negative but there is a localized infection diagnosed using history, examination, and nonmicrobiologic data, these results were defined as “false negative”. “True negative” is regarded as that mNGS is negative and it is concordant with other negative microbiologic tests and fever is attributed to a noninfectious etiology. “Indeterminate” means that the chief physician did not have enough information to adequately adjudicate and classify the case.

# Table S1. The definition and calculation rules of mNGS clinical performance.

|  | Conventional culture test | | | |
| --- | --- | --- | --- | --- |
| mNGS test |  | Positive | Negative | Total |
|  | Positive | A | B | A+B |
|  | Negative | C | D | C+D |
|  | Total | A+C | B+D | A+B+C+D |

Sensitivity: A/(A+C)*100%

Specificity: D/(B+D)*100%

Positive prediction value: A/(A+B)*100%

Negative prediction value: D/(C+D)*100%

Agreement value: (A+D)/(A+B+C+D)*100%

# Table S2. Clinical features of infection in AL patients with FN with positive mNGS results.

|  | **Tax** | **mNGS_only** | **CMTs_only** | **Both** |
| --- | --- | --- | --- | --- |
| Bacteria | Klebsiella pneumoniae | 7 | 0 | 3 |
| Bacteria | Pseudomonas aeruginosa | 3 | 0 | 1 |
| Bacteria | Stenotrophomonas maltophilia | 3 | 0 | 0 |
| Bacteria | Enterococcus faecium | 2 | 0 | 0 |
| Bacteria | Serratia marcescens | 1 | 0 | 1 |
| Bacteria | Klebsiella variicola | 1 | 0 | 0 |
| Bacteria | Escherichia coli | 0 | 0 | 1 |
| Bacteria | Citrobacter freundii | 1 | 0 | 0 |
| Bacteria | Enterobacter cloacae complex sp. | 1 | 0 | 0 |
| Bacteria | Acinetobacter baumannii | 0 | 0 | 1 |
| Bacteria | Citrobacter koseri | 1 | 0 | 0 |
| Bacteria | Enterococcus faecalis | 1 | 0 | 0 |
| Fungi | Aspergillus flavus | 1 | 0 | 0 |
| Viruses | Human betaherpesvirus 5 | 1 | 0 | 0 |

Reference

1 Benamu, E. *et al.* Plasma Microbial Cell-free DNA Next-generation Sequencing in the Diagnosis and Management of Febrile Neutropenia. *Clin Infect Dis* **74**, 1659-1668, doi:10.1093/cid/ciab324 (2022).
